# Sight or smell? Behavioural and heart rate responses in subordinate rainbow trout exposed to cues from dominant fish

**DOI:** 10.7717/peerj.1169

**Published:** 2015-08-06

**Authors:** Johan Höjesjö, Michael Axelsson, Ronja Dahy, Lena Gustavsson, Jörgen I. Johnsson

**Affiliations:** Animal Ecology, Department of Biological and Environmental Sciences, University of Gothenburg, Gothenburg, Sweden

**Keywords:** Salmonids, Physiology, Heart rate, Behavioural, Dominance, Communication

## Abstract

Many animals, including fish, can utilize both vision and the chemical senses in intra-specific communication. However, the relative influence of these sensory modalities on behavioral and physiological responses in social interactions is not well understood. The aim of this study was therefore to investigate the relative effects of visual and chemical stimuli from dominant individuals on the behavioral and physiological responses of subordinate rainbow trout (*Oncorhynchus mykiss*). External electrodes were used to detect ECG signals from free-swimming fish. This method allowed the simultaneous recording of behavioral and physiological responses, and possible sex differences in these responses were also investigated. The results suggest that, in this context, visual cues are more important than chemical cues in settling the social hierarchy in rainbow trout because a combination of chemical and visual exposure generally yielded a response in focal fish that was similar to the response elicited by visual exposure alone. Both activity and physiological responses were most pronounced during the first ten seconds after exposure, with subordinate fish moving closer to the dominant, accompanied by a strong bradycardic response. Furthermore, females acted more boldly and moved closer to the dominant fish than males, but here the effect of the modes was additive, with a stronger effect of the combined visual and chemical exposure. Overall, the extra information furnished to the fish in the form of chemical cues did not change either the behavioral or the physiological response. This result suggests that visual cues are more important than chemically mediated ones for social communication and individual recognition in rainbow trout.

## Introduction

An important aspect of animal communication is the ability to send and receive information about social status (i.e., resource holding potential, defined as an individual’s ability to win or persist in a fight, Enquist & Leimar, 1983; Hurd, 2006; Parker, 1974). This ability is critical for reducing costs of conflicts over resources or over dominance positions in hierarchically structured groups (Huntingford & Turner, 1987). The resource holding potential of an opponent can be inferred by assessment of status-related traits such as body size, weaponry or badges (Bradbury & Vehrencamp, 1998) or by observing the opponents’ success in contests against other individuals (Johnsson & Åkerman, 1998a; Oliveira, McGregor & Latruffe, 1998). Furthermore, to avoid repeated costly interactions with the same dominant/subordinate individual, it is adaptive to be able to identify and subsequently recognize the other individual (Johnsson, 1997; Höjesjö, Johnsson & Axelsson, 1999). In addition, general winner and loser effects tend to conserve relative social status, as contest winners tend to continue their winning streaks, all other things being equal, whereas losers tend to go on losing (Dugatkin, 1997; Schuett, 1997). The communication of relative fighting ability and subsequent maintenance of social status in animal groups can be mediated by a variety of sensory modes depending on the particular animal taxon in question (Alcock, 2005).

Fish, the most species-rich of all vertebrate taxa, have evolved a variety of modes for communication, including visual, chemical, acoustic, mechanic and electrical sensory systems (Rosenthal & Lobel, 2006). Generally, chemical cues in aquatic systems are argued to be of particular importance to mediate communication because water is an excellent medium to transmit a wide range of chemicals and, additionally, because visual cues are disrupted by light attenuation, turbidity, and/or habitat complexity (Ward & Mehner, 2010; Giske, Huse & Fiksen, 1998). Accordingly, many fishes rely on chemical cues for foraging (Webster et al., 2007), mate choice (Aeschlimann et al., 2003) and migration (Hasler & Cooper, 1976). Visual cues, however, may be more important during the formation of stable social hierarchies within species because, especially over shorter distances, visual cues might more directly reflecting an individual’s competitive ability and status (Höjesjö et al., 1998; O’Connor, Metcalfe & Taylor, 1999; O’Connor, Metcalfe & Taylor, 2000). Nevertheless, it has also been argued that chemical cues are used to recognize dominance initially and Barata et al. (2007) have demonstrated how dominant males of the Mozambique tilapia store urine and actively release it during aggressive disputes. Most likely, the urine acts as a ‘dominance’ pheromone to modulate aggression and thereby contribute to social stability.

Salmonid fish, the focus of this study, have excellent vision and chemical senses that are used for a multitude of important tasks. For example, young brown trout (*Salmo trutta*) find cryptic prey (Johnsson & Kjällman-Eriksson, 2008) and avoid predators (Griffiths et al., 2004) by visual detection. Pacific salmon (*Salmo salar*) learn both to recognize their home river (Crapon de Caprona, 1982; Quinn & Dittman, 1990) and assess predation risk (Martel & Dill, 1993) based on chemical cues. As in many other species, visual cues (i.e., body size) are reliable predictors of the outcome of dyadic contests in salmonids (Johnsson, Winberg & Sloman, 2006). Moreover, the finding that contest intensity decreases as opponent size differences increases in dyadic contests suggests that visual cues are used to assess fighting ability (Johnsson, Nöbbelin & Bohlin, 1999). It is not known to what extent salmonids also use chemical cues to communicate intrinsic fighting ability and social status. However, chemical cues have been found to affect aggression levels in groups of Atlantic salmon (Griffiths & Armstrong, 2000).

The behavioral and physiological responses to assessment and contest situations in animals are intrinsically linked (Johnsson, Winberg & Sloman, 2006), and it is well established that threatening situations can induce cardioventilatory responses. In mammals, bradycardia (decreasing heart rate) is associated with freezing or hiding, whereas tachycardia (increasing heart rate) is associated with flight or defense (Belkin, 1968; Moen et al., 1978; Smith, Johnson & Martin, 1981; Espmark & Langvatn, 1985). Furthermore, salmonids respond to predator attacks with bradycardia and flight, followed by a tachycardic response that increases the supply of oxygen to the body as part of the fight or flight response (Höjesjö, Johnsson & Axelsson, 1999; Johnsson, Höjesjö & Fleming, 2001). Furthermore, we have shown that rainbow trout bystanders react rapidly, with behavioral avoidance, to potential opponents. That these opponents later become dominant suggests that bystanders can rapidly assess the fighting ability of potential opponents (Höjesjö et al., 2007). This behavioral effect was subsequently (the following day) followed by an elevated heart rate in bystander fish exposed to dominant opponents. However, the experimental design did not allow us to tease apart the relative effects of visual and chemical stimuli on these responses, and the determination of these effects furnished the main rationale for performing this study.

Accordingly, in the present study, we aim to evaluate the relative effects of visual and chemical stimuli from dominant individuals on the behavioral and physiological responses of subordinates. We also investigate whether males and females differ in this response. Such sex-specific data are presently missing, and our results could, hopefully, provide new important insights. For this purpose, dyadic contests were staged between rainbow trout (*Oncorhynchus mykiss*), and the winner (dominant) and loser (subordinate) were determined. Subordinate individuals were then subjected to visual and/or chemical cues from the dominant, and their behavioral and physiological (i.e., heart rate) response to these cues were recorded and compared with a control (no cues) treatment.

## Methods

The study was performed between February and April of 2007 with one-year-old (1 +) hatchery-reared (Antens Laxodling AB) rainbow trout and approved by the Ethical Committee for Animal Research in Göteborg (license 199/2002). The fish (*n* = 200) were transported from the hatchery in specially designed fish transport containers (stainless steel construction, approximately 1.5 m^3^ in size with built-in aeration). The transport time was less than 40 min, and the density of the fish in the containers complied with the Swedish regulations for transportation of fish.

**Figure 1:**
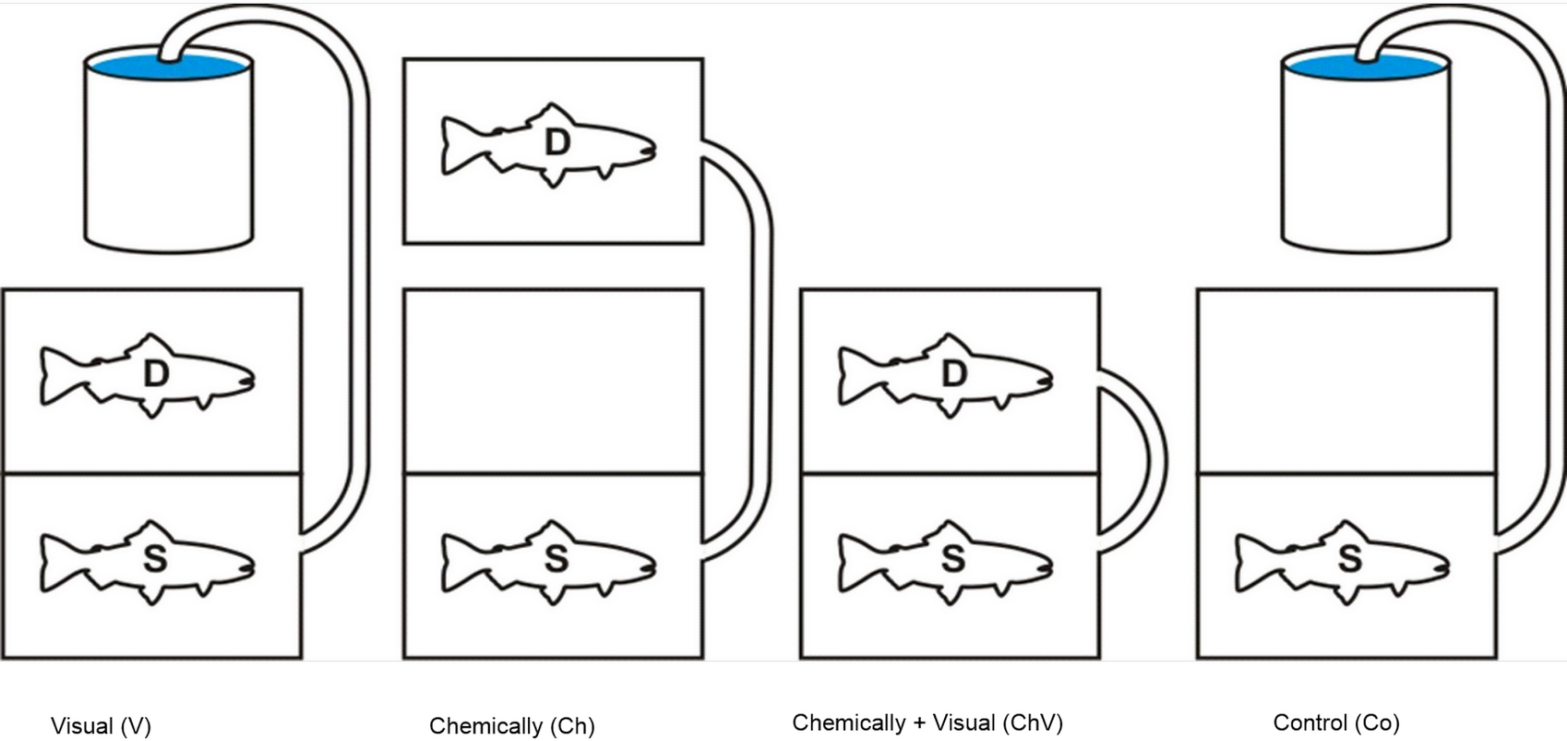
An illustration of the four different treatments used in the study. The subordinate fish (S), our focal fish, is always positioned in the front section, and the dominant (D) is positioned in the back or in a separate aquarium to expose the focal fish only to chemical cues. Visual and chemical cues could be separated through the use of the hatch either separately or in combination with the hose pumping water into the front tank. The chemical cue was always introduced along the longside in the front tank facing the other aquarium in order to standardize the source of the cue.

After arrival at the Department of Biological and Environmental Sciences in Gothenburg, all fish were transferred and kept in large plastic tanks (150 × 75 × 75 cm, h/w/l) for at least 48 h before the trials to acclimatize the animals. Food was provided ad libitum. These tanks were equipped with a floating plastic cover to provide shelter and shadow, thereby reducing the overall stress on the fish. All aquaria used for the experiment, as well as the acclimatization tanks, were supplied with well-aerated water from the main aquarium system. This system used a sand filter and a UV filter. Two types of aquaria were used in the study: “dyadic aquaria” (35 × 34× 64 cm, h/w/l), where the social rank was determined, and “experimental aquaria” (40× 48× 64 cm, h/w/l), where the subordinate fish were exposed to a combinations of cues from the dominant fish. Both “dyadic” and “experimental” aquaria were filled with water (10 °C ± 1) to an approximate depth of 20 cm. The experimental aquaria had a flow through rate of 1.4 l/min. In total, seven experimental aquaria and seven dyadic aquaria were used. In addition, we used two smaller aquaria (20 × 24× 32 cm h/w/l) to transfer chemical cues to the experimental aquaria during the application of the chemical treatment (Fig. 1).

### Dyadic contests

Trout to be used in the experiment were first anesthetized with 2-phenoxy ethanol (0.5 ml/l), measured in weight and length and then transferred to the smaller “dyadic aquaria” where they were allowed to settle for 24 h. Each pair was size-matched on the basis of length (±0.5 cm) to minimize any size-related behavior differences (Huntingford et al., 1990). This aquarium was divided in two parts by placing a dark opaque sliding hatch in the middle. After 24 h, the opaque sliding hatch was carefully hoisted by pulling on a string attached to the hatch. This procedure was used to minimize stress. The social rank of the two fish was then determined. The contests were allowed to last for a maximum of 2 h and lasted, on average, 30 min; the duration of the contests ranged between 20 and 110 min. The dominant fish was defined as the individual winning more than 90% of the interactions (Höjesjö et al., 2007) and retaining its original coloration (O’Connor, Metcalfe & Taylor, 1999). The dominant fish also spend more than 75% in the middle of the aquarium (Höjesjö et al., 2007). Accordingly, we did not observe any abnormal behavior or any injury (defined as visible wounds) to any of the fish during the experiments. No additional refuge was given in the dyadic aquaria because the provision of a refuge would, most likely, have increased the time needed to determine rank, and the subordinate fish were removed as soon as the rank was settled. Hence, the fish were not exposed to more stress than was absolutely needed to assess their relative social rank. After the dyadic contest, opponents were separated by lowering the hatch, and both fish were transferred to either the experimental aquarium or the smaller aquarium used to collect chemical cues and left to acclimate for approximately 4 h. When placed in the experimental aquarium, the subordinate fish was transferred to the front portion of the aquarium and the dominant to either the back portion of the experimental aquarium or the smaller separate aquarium, depending on the treatment. For visual and visual/chemical treatment, the dominant fish (the winner) was placed in the rear section (see Fig. 1).

Although we cannot rule out the possibility of rank reversal in this study, differences in the behavior of many animals (temperament) have been described as both consistent (Reale et al., 2007) and repeatable (Bell, Hankison & Laskowski, 2009). Hierarchies is also, usually stable when they are determined since they are correlated with fighting ability which leads to the phenomena that winner tends to go on winning and losers losing (Abbott & Dill, 1985; Dugatkin & Druen, 2004). This has also been showed in killifish, (McGhee & Travis, 2010) where males showed repeatable linear dominance hierarchies. Over time, there is the possibility off course during ontogenetic development that social status can change due to random effect or difference in habitat quality. During the time frame in this study though, we believe that the social rank is set and stable.

### Visual and chemical exposure

The experimental aquarium was divided into two parts, with sealed, transparent Plexiglas in the middle of the aquarium. The Plexiglas divider allowed no water to circulate between the two sections. In addition, an opaque sliding hatch next to the transparent Plexiglas prevented the fish from seeing each other. Strings were attached to the top of the opaque sliding hatches, allowing them to be carefully hoisted upward and downward without stressing the fish. The subordinate, our focal fish, was placed in the front section (Fig. 1) and the dominant placed in the back section. The placement of the front and back sections alternated between the left- and right-hand sides of the experimental aquarium. The dominant fish could also be placed in one of the smaller aquaria if only the effect of chemical exposure were being tested.

The fish were chemically isolated from each other by a separate in- and outflow of water in each section. A silicon tube connected to a peristaltic pump (built to order by RS Components) was attached to the aquarium, allowing water from various sources to be pumped (120 ml/min) into the compartment holding of the subordinate individual. The water could be pumped from the section containing the dominant fish or from an aquarium containing no fish, depending on the treatment. The section with the focal fish (subordinate) also had an electrode cage (see details below) positioned in that section, allowing non-invasive measurements of the heart rate of the fish (Höjesjö et al., 2007; Goodman & Weinberger, 1971; Altimiras & Larsen, 2000). Hence, by having the two fish completely chemically isolated from each other, we could use the hatch with only visual exposure and/or in combination with a separate chemical exposure. The following four treatments were used:

(1) Visual exposure, (2) Chemical exposure, (3) Visual and chemical exposure, (4) Control with only exposure of water and the sight of an empty compartment to control for any effect of disturbance during the actual procedure (Fig. 1).

To avoid disturbing the fish, we observed them through a hole in a plastic cover for two minutes before and five minutes after the opaque sliding hatch was hoisted and/or the addition from the tube with chemical cues/fresh water. The section containing the focal fish was divided into nine horizontal, virtually equally sized subsections (1–9) according to a one-dimensional coordinate system, with subsection nine being situated in front of the tank closest to the dominant fish. An index was then calculated based on the position of the focal fish. A greater index value corresponded to a shorter distance relative to the experimental tank occupied by the dominant individual. Activity was also observed and scored using the following index: 1, Holding bottom (e.g., some part of the fish touching the bottom); 2, Holding low; 3, Holding high (the majority of the fish positioned in the upper half of the water column); and 4, Swimming. The position and activity of the fish were recorded every 10 s and used to assess the change in movement and activity over time when exposed to a dominant individual. To examine any effects of habituation, the responses were recorded during three subsequent observations at approximately 4 h (Obs. 1), 6 h (Obs. 2) and 28 h (Obs. 3) after the rank was settled.

In total, we tested 196 (98 pairs) fish, but only fish that could be ranked clearly were used in the study. The result was that a total of 83 subordinate fish were exposed to either visual cues (*n* = 23) chemical cues (*n* = 20), visual and chemical cues (*n* = 21) or controls (*n* = 19). The order of the treatments was randomized, and all fish were tested only once. After the last observation, all focal fish were euthanized with an overdose of 2-phenoxyethanol and a lethal blow to the head, weighed and sexed by examination of the gonads. Dominant fish were not killed, and therefore no data are available on their sex.

### Heart rate monitoring

Essentially the same setup as in Höjesjö, Johnsson & Axelsson (1999), Höjesjö et al. (2007) was used in the present study. In short, the setup consists of stainless steel electrodes for detecting bioelectric potentials in the water generated by the active muscles of the fish. From the raw signal, the ECG can then be separated by careful filtering. A grid placed on the bottom of the chamber acts as one of the electrodes, and a rectangular cage of fine stainless steel wires (2 mm diameter) placed immediately below the water surface constitutes the other electrode (Höjesjö et al., 2007). A common electric ground electrode bar is placed in the surrounding water. The fish could move freely between the two electrodes and the electric ground electrode bar without noticeably affecting the quality of the signal. The raw signals were amplified using four BIO Amplifiers (model ML136, ADInstruments, Castle Hill, Australia). All recordings were saved at a frequency of 200 Hz in the EEG mode of the BIO Amplifier, which was pre-set to the following configuration: range: EEG mode, 1 mV; low-pass filter: 120 Hz; high-pass filter: 1 s; 50 Hz notch filter activated. The signals from the BIO Amplifiers were then passed to a PowerLab 8/30 system (ADInstruments, Castle Hill, Australia), and data were collected on a PC with ADInstruments acquisition software LabChartTM 7 Pro v7.3.7. In LabChart, the signal was further filtered and processed off-line to separate the electrocardiogram (ECG) signal from other muscular activity, resulting in data on heart rate over time (average beat min^−1^ during 10 s intervals based on the time between beats).

### Data analysis

For the behavioral data, to focus on the event of exposure, ten 10 s periods before the opaque sliding hatch was hoisted and ten 10 s periods after the opaque sliding hatch was hoisted were analyzed (a total of 200 s of data). The average heart rate for every 10 s period was calculated and synchronized with the behavioral data. However, because the change in heart rate is most likely a more rapid response (see Höjesjö et al., 2007 and references within), we only used five 10 s intervals before and five 10 s intervals after the exposure for analyses. The three dependent variables, e.g., activity, position and heart rate, were all normally distributed. Therefore, separate repeated measures analyses of covariance (Systat 11, Richmond, California, USA) were used with time (the 20 time periods for the behavioral data and 10 time periods for the heart rate data) as the repeated measures factor, treatment (chemical, visual, chemical + visual, control) and sex (males and females) as independent class variables and weight (g) as a continuous covariate. Interactions between time and treatment, as well as between sex and treatment, were initially included in the model but were removed using a stepwise procedure if not significant. (Activity/Position/Heart rate = Time + Treatment + Sex + Weight + Time × Treatment + Sex × Treatmnet) To clarify the presentation of the results, only *p* values less than 0.10 are presented.

## Results

Overall, 83 fish (average size; 23.3 cm ± 2.8 stdev, 142.7 g ± 47.8 stdev) were analyzed in the study in terms of behavioral responses (activity and position). These 83 fish comprised 45 males and 36 females (and 2 fish whose sex could not be determined), evenly distributed among treatments (Control: 8 female, 10 males; Chemical: 7 female, 13 males; Visual: 12 female, 11 male; Chemical + Visual: 9 female, 11 males). For heart rate measurements, all fish from which no signal could be accurately filtered were removed, leaving 42 individuals to be analyzed for HR (Control: 3 female, 7 males; Chemical: 5 female, 6 males; Visual: 6 female, 4 male; Chemical + Visual: 5 female, 6 males)). There were no differences in size among treatment groups or between sexes (*p* > 0.1 both cases).

### Position

**Figure 2:**
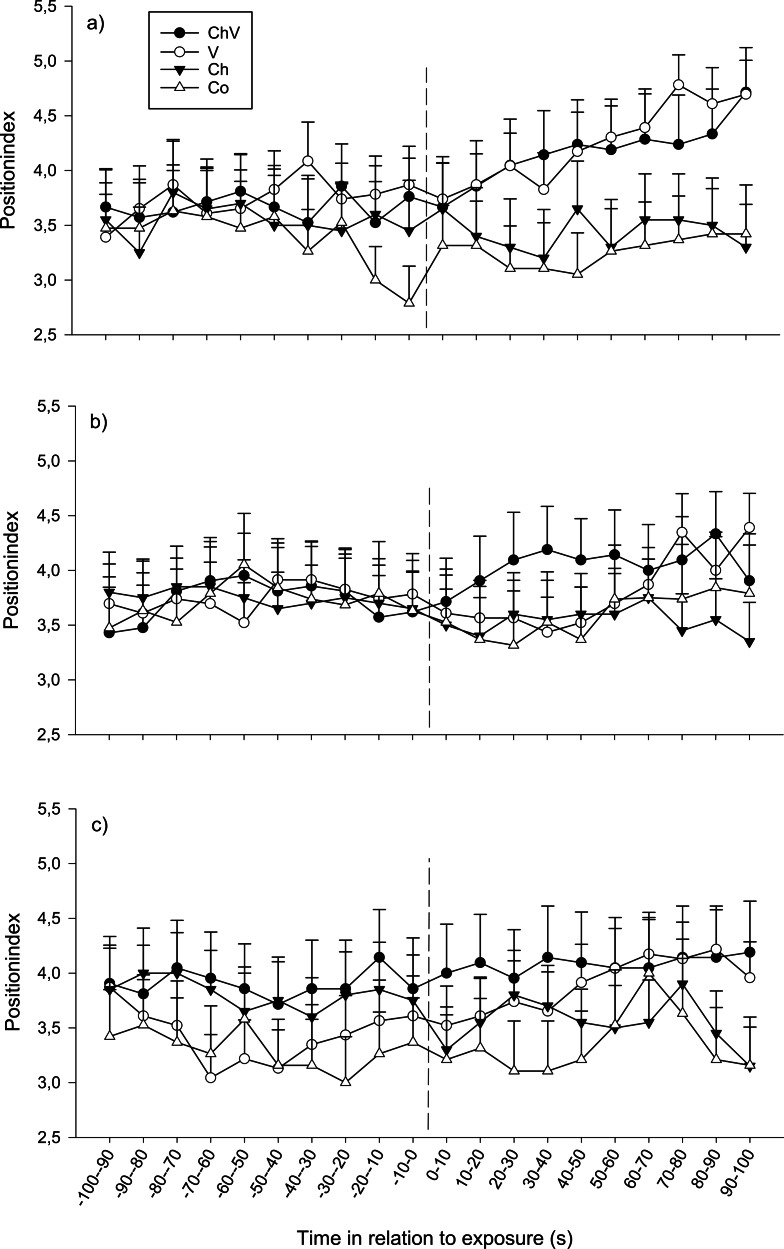
The change in position of the subordinate fish during the first (A), second (B) and last (C) observation for the different treatments (ChV, Chemical and Visual; V, Visual; Ch, Chemical and Co, Control). The dotted line refers to the time of exposure. Error bars denote the standard error of the group means. Here, only positive values of the standard error are presented.

At the first observation, there was a significant interaction between time and treatment (*F*_57,1368_ = 1.40, *p* = 0.028; Fig. 2A). The reason for this interaction was that only visual or a combination of visual/chemical cues caused the focal fish to move closer to the dominant fish, whereas control and chemical cues did not cause any change in position. At the second observation, all fish moved closer to the dominant fish regardless of treatment (*F*_19,1368_ = 1.82, *p* = 0.017, Fig. 2B). Neither a significant effect of treatment nor any interaction could be detected at the third observation. Furthermore, there was no difference in position due to weight or length.

**Figure 3:**
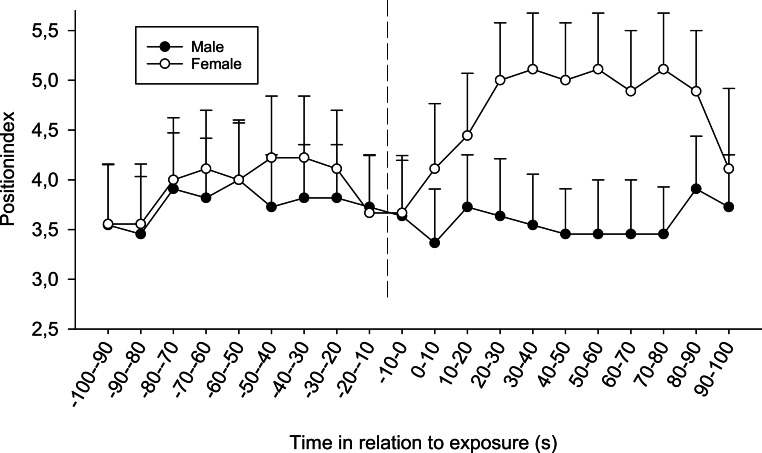
The difference between sexes in the position index for fish exposed to both visual and chemical cues (chemical + visual treatment) during the second observation. The dotted line refers to the time of exposure. Error bars denote the standard error of the group means. Here, only positive values of the standard error are presented.

There were also differences between the sexes; regardless of time and treatment, females tended to be positioned closer to the opaque sliding hatch than males during the first (*F*_3,72_ = 2.21, *p* = 0.094) and significantly closer during the second exposure (*F*_1,72_ = 4.24, *p* = 0.043, position index; 4.14 ± 0.23 SE for females and 3.42 ± 0.23 SE for males ). Furthermore, for the second observation, it was obvious from the separate results from the four treatments that only females in the combined chemical/visual exposures increased their position indexes, i.e., moved closer to the dominant fish (Friedman *p* < 0.01, Friedman test statistic = 50.20), whereas males did not (Fig. 3).

### Activity

**Figure 4:**
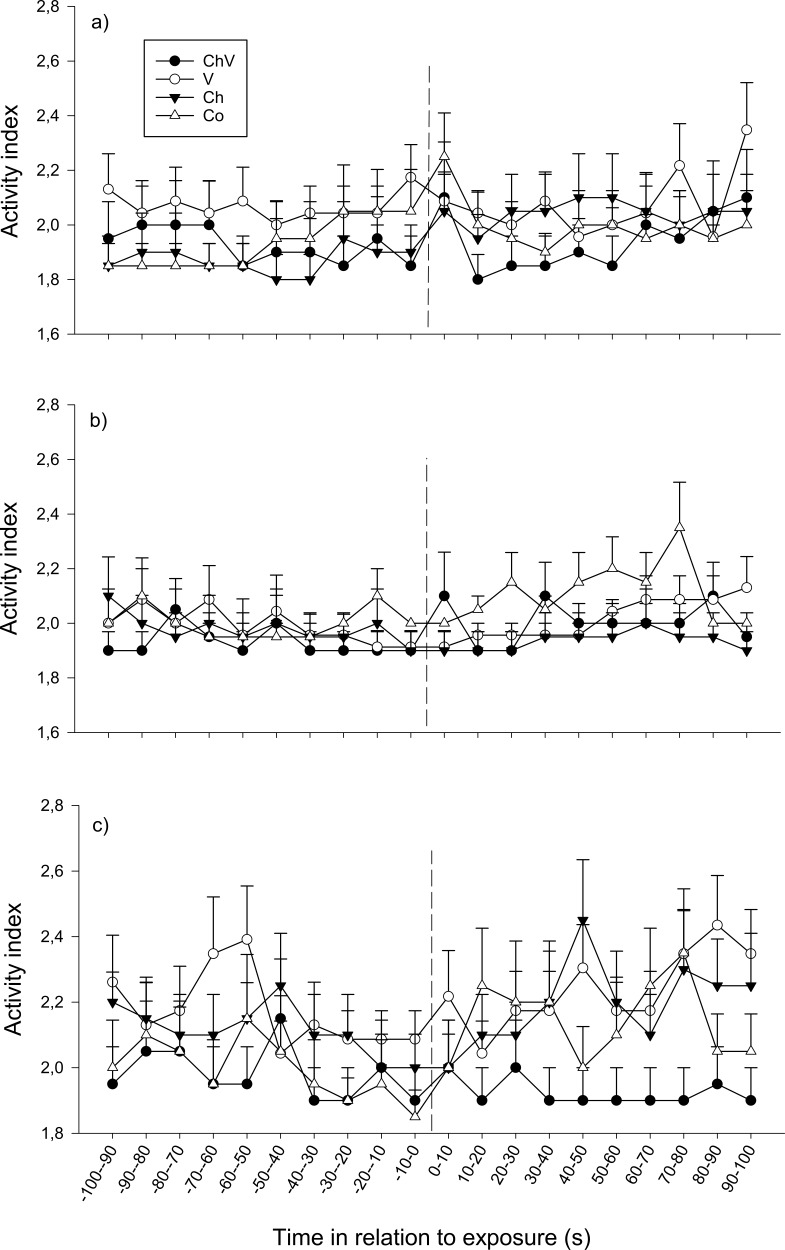
The change in activity of the subordinate fish during the first (A), second (B) and last (C) observation for the different treatments (ChV, Chemical and Visual; V, Visual; Ch, Chemical and Co, Control). The dotted line refers to the time of exposure. Error bars denote the standard error of group means. Here, only positive values of the standard error are presented.

The overall activity did not change over time for any of the three observations (*p* > 0.1 exposure 1) even if there was a tendency for activity to differ among treatments over time on the second (*F*_57,1368_ = 1.288, *p* = 0.076, Fig. 4B) and third (*F*_57,1368_ = 1.324, *p* = 0.056, Fig. 4C) observation. At the second observation, this result most likely occurred because the fish in the control treatment increased their activity, whereas the fish from the other treatment groups remained at a lower activity level (Fig. 4B). Similarly, at the third observation, the fish in the chemical/visual treatment tended to decrease their activity after exposure, whereas the fish in the other treatments appeared to increase their activity (Fig. 4C). In addition, treatment tended to have a general effect on activity at the third observation (*F*_3,72_ = 2.29, *p* = 0.085), whereas fish in the chemical/visual treatment appeared to have a lower activity level (Fig. 4C).

There were no effects of sex during the first two observations, but there was a significant interaction between sex and treatment at the third observation (*F*_3,72_ = 9.93, *p* = 0.031), when females exposed to chemical/visual cues remained at a relatively low activity level (average activity index after exposure 1.7 ± 0.18) compared with female fish exposed to any of the other three treatments (activity index for females exposed to visual cues: 2.25 ± 0.12 SE, chemical cues: 2.27 ± 0.15 SE and for control: 2.39 ± 0.19 SE). In males, no clear response in activity could be detected, neither an overall response nor any difference between the treatments.

### Heart rate

**Figure 5:**
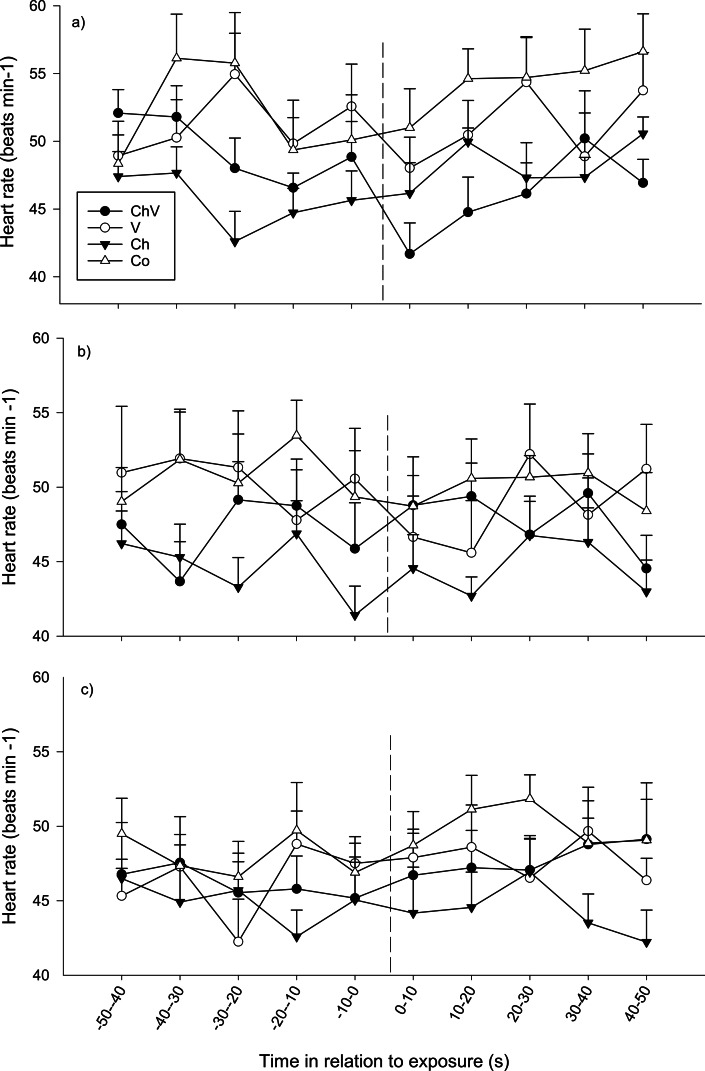
The average change in heart rate (±SE) of the subordinate fish during the first (A), second (B) and last (C) observation for the different treatments (ChV, Chemical and Visual; V, Visual; Ch, Chemical and Co, Control). The dotted line refers to the time of exposure. Error bars denote the standard error of group means. Here, only positive values of the standard error are presented.

During the first observation, there were no overall differences between treatments nor any general change in heart rate over time (*p* > 0.1 all cases). However, a significant interaction between time and treatment was found, indicating that the change in heart rate over time differed between treatments (*F*_27,130_ = 1.613, *p* = 0.03; Fig. 5). Fish in the visual/chemical and visual treatment responded with bradycardia (decreasing heart rate) immediately after exposure (first 10 s interval), whereas heart rate in the chemical and control treatments remained unaffected. During the second observation, heart rate decreased over time (*F*_9,288_ = 2.268, *p* = 0.018) after exposure regardless of treatment (*p* > 0.1, Fig. 4). No significant differences were found at the last observation (*p* > 0.1).

**Figure 6:**
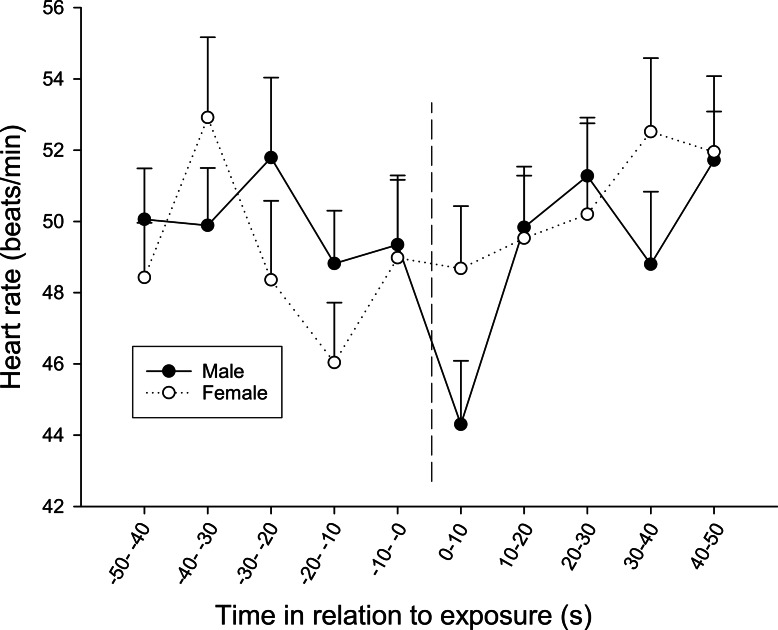
The overall difference in heart rate response between sexes during the first observation. The dotted line refers to the time of exposure. Error bars denote the standard error of the group means. Here, only positive values of the standard error are presented.

At the first observation, there was also a significant interaction between time and sex (*F*_9,270_ = 1.920, *p* = 0.049), where males showed a bradycardic response after the exposure, a response that was lacking in females (Fig. 6).

## Discussion

In this study, we found that a combination of chemical and visual exposure generally yielded a response in focal fish similar to that resulting from visual exposure alone. This finding suggests that within this context, visual cues are more important than chemical cues for recognizing and responding to individuals within a social hierarchy in rainbow trout. Both activity and physiological responses were generally immediate, occurring within 10 s following the first exposure. The position response was positive, with the focal fish moving closer to the dominant, most likely a type of inspection behavior (Dugatkin & Godin, 1992), whereas the change in activity was more subtle and less immediate. Fish exposed to both chemical and visual cues reduced their activity after the second and last exposure and, in addition, the general overall activity was lower at the last exposure. Similarly, the heart rate response was rapid during the first observation, where fish exposed to the combined visual/chemical and visual treatment responded with a bradycardia after the exposure. Hence, the extra information provided when adding chemical cues did not change the observed behavioral or physiological responses.

The literature presents conflicting findings on the relative importance of visual and chemical cues in fish. It is generally suggested that visual communication may be more accurate, direct and reliable and, therefore, plays a larger role in individual recognition, which is crucial for the establishment of social hierarchies. In contrast, chemical cues most likely occur in a more diffuse context and will mix with chemicals originating from different individuals as well as compounds originating from other species and various processes within the ecosystem (Bradbury & Vehrencamp, 1998; McLennan, 2003). Nevertheless, chemical signals have been found to be an effective means of communication in a wide range of taxa in the contexts of predator avoidance (Brönmark & Hansson, 2000; Brown & Chivers, 2006; Dahl, Nilsson & Pettersson, 1998), foraging (Fine, Winn & Olla, 1997; Hara, 1993; Ringler, 1979; Glova, Sagar & Naslund, 1992) migration (Sorensen et al., 2005; Crapon de Caprona, 1982), shoaling (Wisenden et al., 2003), kin recognition (Brown & Brown, 1993; Griffiths, Armstrong & Metcalfe, 2003) and reproduction (Belanger & Moore, 2006; Díaz & Thiel, 2004). These are all examples of communication in a context that does not require individual recognition.

Visual cues, however, may allow recognition of specific individuals and could, therefore, be important for assessing and remembering the competitive ability of conspecifics, thereby avoiding costly conflicts in which the probability of winning is low (Enquist & Leimar, 1983; Johnsson & Åkerman, 1998a; Johnsson & Åkerman, 1998b). It is well known that subordinate salmonids darken during social interactions (Keenleyside & Yakamoto, 1962), a response that is believed to act as a signal modifying social behavior, thus reducing costly interactions. Similarly, dominant individuals within a species (this study, Keenleyside & Yakamoto, 1962; Höjesjö et al., 1998; Höjesjö et al., 2007) generally signal their superior status with erect fins and brighter coloration (O’Connor, Metcalfe & Taylor, 1999). Smith & Belk (2001) have suggested that during more risky behaviors such as predator inspection, mosquitofish (*Gambusia affinis*) rely mainly on visual cues, whereas general avoidance behavior is determined by additive responses from visual and chemical cues. This generalization is supported by the findings of our study. We found that a visual stimulus from a dominant conspecific causes a larger response in the subordinate focal fish and that no additive response of the chemical stimuli could be detected. Alternatively, the subordinate individual might not have had the opportunity to learn the chemical cues of a specific dominant within the given time frame. In addition, dyadic and focal testing was conducted in relatively small tanks, and this close proximity might result in a ‘magnification’ of the relative value of visual information. Hence, in a more realistic natural environment, chemical cues may function as a first cue that can alert the receiver to the presence of a second visual cue, thereby increasing the probability of detection and recognition by the receiver (e.g., Smith & Belk, 2001). There are several examples from various taxa suggesting that chemical cues are less species- and/or individual-specific compared with visual cues and may not transfer the same amount of information but may be used complementarily in the field (McLennan, 2003; Kim et al., 2009; Palaoro, Ayres-Peres & Santos, 2013) and may be used to enhance the accuracy with which receivers assess a single quality (e.g., Johnstone, 1996). However, Brown & Magnavacca (2003) suggest that chemical cues, in the form of prey alarm cues in the diet of the predator, are the primary source of information regarding local predation risk during inspection behavior and that visual cues are used if chemical information is unavailable or ambiguous. Bro-Jørgensen (2010) very nicely illustrates how dynamic selection, in a fluctuating ecological and social environment, can explain why multiple signals can be used to convey a message. Although visual cues are shown to be more important in this study, information transfer between individuals may, of course, be context dependent; in other situations, such as those involving reduced visibility, chemical cues may play a larger and/or additive role. In a natural river, chemically mediated cues might also be more predictable and mainly orientated according to a unidirectional flow from an upstream source.

In agreement with previous studies, our data verify that the behavioral and physiological (change in heart rate) responses are linked (Höjesjö, Johnsson & Axelsson, 1999; Johnsson, Höjesjö & Fleming, 2001). The physiological response of the focal fish also suggests that the information is mediated primarily by visual cues, at least in the short time frame, when the focal fish expressed a bradycardiac response. In a previous study (Höjesjö et al., 2007), the overall heart rate increased for focal fish sharing water with a superior contestant after 24 h, whereas no such difference could be detected in the current study. In the current study, however, the chemical cues associated with the dominant fish were separated from the subordinate fish. Nevertheless, only the combination of chemical and visual cues or the visual cue alone resulted in the observed bradycardiac response, suggesting that visual cues are also the most important for generating a physiological response.

The more delayed tachycardic response found in the previous study cited above most likely arose from the more permanent chemical cues associated with the presence of a dominant opponent or a combination of initial visual cues and subsequent continuous exposure to chemical cues. Physiological responses towards stress such as the presence of a dominant individual are relatively well documented in teleost fish (see reviews by Pickering & Pottering, 1995), and the initial response can be a first rapid bradycardia mediated via the vagal innervation of the heart; this response then reverses and become tachycardia. The physiological significance of fright bradycardia has been discussed since it was first described by Belkin in 1968. It is well known that the electric signals that are generated by active muscles in the fish “leak” out in the water (Kalmijn, 1974; Kalmijn, 1988), and this electric profile can be used by a predator to locate prey (Wueringer et al., 2012). Moreover, alteration of the electric profile can be used to avoid the predator (Kempster, Hart & Collin, 2013). The initial rapid bradycardia observed in this study could be an attempt by the subordinate to mask itself electrically in the water and thereby avoid interaction with the dominant individual.

This study also demonstrates differences between sexes in the response. Females generally acted more bold (moved closer to opponents) and were more active when presented with the combined chemical and visual cue but not with the visual cue alone. Hence, there seemed to be an additive but sex-specific effect of visual and chemical cues, as each of the cues alone did not cause any change in position or activity in females. The bradycardiac response at the first observation when fish were exposed to a combined exposure of visual and chemical cues was also more pronounced in females. This result is somewhat surprising as juvenile male rainbow trout have previously been found to be more aggressive than females (Johnsson & Åkerman, 1998a; Johnsson & Åkerman, 1998b), and similar sex-specific aggression patterns have been reported in brown trout, where males also tend to be more bold than females (Johnsson, Höjesjö & Fleming, 2001). Similarly, Cooke et al. (2004) detected that nesting males of largemouth bass had higher resting cardiovascular rates relative to non-nesting males and females, most likely linked to aggressive nest defense by males, which is critical for brood survival in this species (Sutter et al., 2012). The boldness detected in the females in our study could alternatively be interpreted as a form of inspection behavior (Dugatkin & Godin, 1992; Brown & Magnavacca, 2003) whose overall function would be to avoid costly conflicts in which the probability of winning was low (Enquist & Leimar, 1983). These results are somewhat contradictory to the findings of our previous study (Höjesjö et al., 2007), where male bystanders moved closer to opponents prior to any information on the opponents’ competitive ability. Juvenile sex differences in behavior may be favored as a correlated response to sexually selected genes in adult males and females during reproduction (Cheverud, Rutledge & Atchley, 1983; Bakker, 1994). The present finding is, to our knowledge, one of the few in juvenile fish suggesting that females are more bold and active than males, and the matter needs to be further investigated.

In summary, the subordinate fish in our study showed similar behavioral and heart rate responses to a combination of chemical and visual exposure and visual exposure alone, suggesting that visual cues are more important than chemically mediated ones during the formation and stabilization of social hierarchies in rainbow trout. To our knowledge, this is the first study that has evaluated both behavioral and physiological responses in subordinate fish exposed to both visual and chemical stimuli in a controlled experiment.

## Supplemental Information

10.7717/peerj.1169/supp-1 Supplemental Information 1 Raw data activity

10.7717/peerj.1169/supp-2 Supplemental Information 2 Raw data position

10.7717/peerj.1169/supp-3 Supplemental Information 3 Raw data Heart rate
